# My Creed

**Published:** 1907-06

**Authors:** 


### Abstracts and Selections.

### My Creed.

Do not keep the alabaster boxes of your love and tenderness sealed up until your friends are dead. Fill their lives with sweetness. Speak approving, cheering words while their ears can hear them, and while their hearts can be thrilled and made happier by them; the kind things you mean to say when they are gone, say before they go. The flowers you mean to send for their coffins, send to brighten and sweeten their homes before they leave them.

If my friends have alabaster boxes laid away, full of fragrant perfumes of sympathy and affection, which they intended to break over my dead body, I would rather they would bring them out in my weary and troubled hours, and open them, that I may be refreshed and cheered by them while I need them. I would rather have a plain coffin without a flower, a funeral without an eulogy, than a life without the sweetness of love and sympathy.

Let us learn to anoint our friends beforehand for their burial. Post-mortem kindness does not cheer the troubled spirit. Flowers on the coffin cast no fragrance backward over life's weary way.

Chicago. W. D. Boyce.

---

Treasury Department,
Bureau of Public Health and Marine Hospital Service.

Washington, D. C., May 17, 1907.

A board of officers will be convened to meet at the Bureau of Public Health and Marine Hospital Service, 3 B Street S. E., Washington, D. C., Monday, July 15, 1907, at 10 o'clock a. m., for the purpose of examining candidates for admission to the grade of assistant surgeon in the Public Health and Marine Hospital Service.

Candidates must be between 22 and 30 year of age, graduates of a reputable medical college, and must furnish testimonials from responsible persons as to their professional and moral character.

The following is the usual order of the examinations: 1, physical; 2, oral; 3, written; 4, clinical.

In addition to the physical examination, candidates are required to certify that they believe themselves free from any ailment which would disqualify them for service in any climate.

The examinations are chiefly in writing, and begin with a short autobiography of the candidate. The remainder of the written exercise consists in examination of the various branches of medicine, surgery, and hygiene.

The oral examination includes subjects of preliminary education, history, literature and natural sciences.

The clinical examination is conducted at a hospital, and when practicable, candidates are required to perform surgical operations on a cadaver.

Successful candidates will be numbered according to their attainments on examination, and will be commissioned in the same order as vacancies occur.

Upon appointment the young officers are, as a rule, first assigned to duty at one of the large hospitals, as at Boston, New York, New Orleans, Chicago or San Francisco.

After five years' service, assistant surgeons are entitled to examination for promotion to the grade of passed assistant surgeon.

Promotion to the grade of surgeon is made according to seniority, and after due examination as vacancies occur in that grade.

Assistant surgeons receive $1600, passed assistant surgeons $2000, and surgeons $2500 a year. When quarters are not provided, commutation at the rate of thirty, forty and fifty dollars a month, according to grade, is allowed.

All grades above that of assistant surgeon receive longevity pay, 10 per cent in addition to the regular salary for every five years' service up to 40 per cent after twenty years' service.

The tenure of office is permanent. Officers traveling under orders are allowed actual expenses.

For further information, or for invitation to appear before the board of examiners, address "Surgeon-General, Public Health and Marine Hospital Service, Washington, D. C."

